# Syndromic Surveillance Using Veterinary Laboratory Data: Algorithm Combination and Customization of Alerts

**DOI:** 10.1371/journal.pone.0082183

**Published:** 2013-12-11

**Authors:** Fernanda C. Dórea, Beverly J. McEwen, W. Bruce McNab, Javier Sanchez, Crawford W. Revie

**Affiliations:** 1 Department of Health Management, University of Prince Edward Island, Charlottetown, Prince Edward Island, Canada; 2 Animal Health Laboratory, University of Guelph, Guelph, Ontario, Canada; 3 Animal Health and Welfare Branch, Ontario Ministry of Agriculture Food and Rural Affairs, Guelph, Ontario, Canada; University of Catania, Italy

## Abstract

**Background:**

Syndromic surveillance research has focused on two main themes: the search for data sources that can provide early disease detection; and the development of efficient algorithms that can detect potential outbreak signals.

**Methods:**

This work combines three algorithms that have demonstrated solid performance in detecting simulated outbreak signals of varying shapes in time series of laboratory submissions counts. These are: the Shewhart control charts designed to detect sudden spikes in counts; the EWMA control charts developed to detect slow increasing outbreaks; and the Holt-Winters exponential smoothing, which can explicitly account for temporal effects in the data stream monitored. A scoring system to detect and report alarms using these algorithms in a complementary way is proposed.

**Results:**

The use of multiple algorithms in parallel resulted in increased system sensitivity. Specificity was decreased in simulated data, but the number of false alarms per year when the approach was applied to real data was considered manageable (between 1 and 3 per year for each of ten syndromic groups monitored). The automated implementation of this approach, including a method for on-line filtering of potential outbreak signals is described.

**Conclusion:**

The developed system provides high sensitivity for detection of potential outbreak signals while also providing robustness and flexibility in establishing what signals constitute an alarm. This flexibility allows an analyst to customize the system for different syndromes.

## Introduction

The emergence of new diseases and the increasing threat of bioterrorism have motivated the development, especially since the turn of the century, of surveillance systems focused on the early detection of disease. Early work in the field focused on identifying data that could contain signatures of disease introduction, resulting in the exploration of various data sources registering healthcare-seeking behaviours, such as sales of over-the-counter medicine, emergency hospital visits and laboratory test requests [1]. While these data precede diagnostic confirmation, observations can be aggregated and monitored based on syndrome characteristics; an approach which led to the term “syndromic surveillance” entering the scientific literature [2].

The next steps in syndromic surveillance research focused on the development of detection algorithms [3]. Algorithm development and evaluation took into consideration the specific temporal characteristics of surveillance data, such as daily autocorrelations, seasonal trends and day-of-week effects [4]. This also had to consider the context of any detection, such as the availability of temporal and/or spatial data, the influence of external factors in any particular source of data, or even the availability of multiple, and sometimes conflicting, data streams [5]. This research indicated that different algorithms demonstrate better performance in different scenarios (e.g. different ‘shapes’ of outbreak signal) [3], and efforts are being made to combine approaches, rather than settle on one ‘best’ algorithm [6], [7].

Attention has also been given to the issue of preventing outbreak signals that do occur from reducing the performance of detection algorithms that operate prospectively and in any automated manner. Researchers using data-driven methods have demonstrated that sensitivity of detection can be increased by the use of a “guard-band” between the baseline data and the time point being evaluated, in order to avoid contamination of the baseline with an outbreak signal [4], [5], [8]. Methods for preventing parameters from being automatically updated in case of an alarm, for model-based systems, have also been discussed [9], [10]. However, the use of detection algorithms to remove detected signals from the time series, during automated monitoring, has not to the knowledge of the present authors been discussed.

In previous work we addressed the use of diagnostic test requests made to an animal health laboratory as a syndromic data source, first preparing the data for use [11], [12] and then evaluating the performance of different detection algorithms [13]. The results indicated that Shewhart and Exponentially Weighted Moving Averages (EWMA) control charts, as well as Holt-Winters exponential smoothing, could detect potential outbreak signals in the data with high sensitivity. However, none of these approaches was superior to the others in all scenarios of outbreak signal shape and duration. The previous results also highlighted the need to customize the system for the different time series (i.e. syndromic groups) being evaluated, given the effect of the baseline data on algorithm performance, something also discussed in [14].

In this paper the use of all three algorithms, in combination, is explored. The automated implementation of this approach, including a method for filtering potential outbreak signals after they have been detected, is described. A scoring system to detect and report alarms using these algorithms in a complementary way is proposed. This system also provides robustness and flexibility in the establishment of what signals constitute an alarm. This flexibility also allows an analyst to customize the system for different syndromes.

## Methods

### Data-source

The Animal Health Laboratory (AHL) is a full-service veterinary diagnostic laboratory that serves livestock, poultry and companion animal veterinarians in the province of Ontario, Canada. The laboratory receives around 65,000 case submissions per year, resulting in the execution of over 800,000 individual laboratory tests, of which 10% relate to cattle submissions. Test requests for diagnoses of disease in cattle were monitored at the day of submission – pre-diagnostic.

Syndromic groups were created based on the type of sample submitted and the diagnostic test requested by the veterinarian. A common standard for the classification of syndromes has not been developed in veterinary medicine. Classification was therefore based on manual review of three years of available data, and then creating rules of classification reviewed by a group of experts (a pathologist, a microbiologist and a clinical veterinarian) until consensus was reached by the group. These rules were implemented in an automated system classification as documented in Dórea et al. 2013 [11]. Individual health events were defined as any single syndromic occurrence per herd on a given day. Time series composed of daily counts of events for each specific syndromic group will be referred as “syndromic series”.

Seventeen syndromic groups were defined. Once each test request was classified into a syndromic group, the data were collapsed by the unique herd identifier for each day. Due to a very low number of submissions on weekends, any cases in the database assigned to weekends were summed to the following Monday, and weekends were removed from the data. The goal of the system was to allow data streams to be monitored daily, but only syndromic series with a median greater than one case per day (10 from the total 17) were deemed appropriate for daily monitoring [12]. Two of these are presented here as they help illustrate the methods developed: daily counts of laboratory test requests related to mastitis diagnostics in cattle (*mastitis series*) and for identification of bovine leukemia virus (*BLV series*) [11], [12]. Tests for BLV are often requested in animals with a decrease in body condition as well as milk production. This series was chosen due to the statistical similarities to the time series of other syndromic groups, while being the only times series showing evident presence of potential outbreak signals documented in the historical data. Additionally, the counts of test requests for diagnosis of mastitis are used to illustrate the particular effect of working with time series with stronger seasonal effects. Daily counts of laboratory submissions for diagnostic of respiratory syndromes (*respiratory series*), one of the syndromic groups with the lowest median number of submissions per day, are also presented.

Data from 2008 and 2009 were used as training data. These data had been previously analysed in order to remove potential outbreak signals and excessive noise, creating *outbreak-free baselines* for each syndromic series [12]. Untreated data from 2010 and 2011 were used to evaluate the methods described.

### Simulated data

The data simulated in a previous study, which evaluated the performance of each detection algorithm individually [13], was also used to evaluate the performance achieved by combining algorithms in this study. These data were simulated using a Poisson regression model with variables to account for day-of-week and month to reproduce the normal behaviour of the baseline series. The choice of a Poisson regression was based on the result of retrospective analyses of the data [12]. The predicted value for each day of the year was set to be the mean of a Poisson distribution, and this distribution was sampled randomly to determine the value for that day in a given year, for each of 50 simulated years. Outbreak signals were then injected simulating five different shapes (single spike, moving average, linear increase, exponential increase and lognormal increase), four magnitudes (one to four times the baseline counts) and three lengths to peak (one, two and three weeks). Several simulated time series were generated, each containing only one specific outbreak type, repeated over 200 times, and separated by at least 70 days of non-outbreak data (to ensure separation greater than the baseline window of 50 days used). Details are described in [13].

The rationale of simulating five different outbreak shapes comes from the uncertainty regarding how an outbreak in the field would translate into an outbreak signal in the laboratory data. The use of submissions assumes that the epidemiological unit is a herd (not an animal), and it is hard to predict what percentage of the infected herds would be included in the catchment population of the data source monitored. These issues have been discussed when the simulated data were presented in the first part of this work [13]. *Due to these uncertainties, a range of outbreak signal shapes previously documented in simulation studies for development of syndromic monitoring were reproduced in this study* [15], [16].

Only the days up to an outbreak peak were simulated, as our aim was to provide a comparison of how quickly algorithms would detect an outbreak signal, *before* its peak. Outbreaks that were not detected by their peak were considered to be undetected.

### Algorithms for detection of outbreak signals

Based on previous quantitative evaluations [13], using the actual syndromic series as well as simulated data with controlled injection of outbreaks, three detection algorithms were selected with the following detection settings:

Exponentially Weighted Moving Averages control charts (EWMA) with a smoothing parameter of 0.2, baseline of 50 days, guard-band of 10 days (time between the point being evaluated and the baseline), and a detection limit of 2 standard deviations.
Shewhart control charts with a guard-band of 10 days, baseline of 50 days, and detection limit of 2.25 standard deviations.
Holt-Winters exponential smoothing (HW) with a baseline of 2 years, and detection limit based on the upper limit of the 97.5% confidence interval for model prediction; using 5-day-ahead predictions (guard-band).

The control charts were applied to data pre-processed by weekly differencing, while the HW method was applied to data directly.

### Automated filtering potential outbreak signals

In order to preserve an outbreak-free baseline when prospective (“on-line”) monitoring was implemented, automated filtering of the potential outbreak signals was necessary. In order to make the filtering mechanism applicable to all algorithms being tested, a correction value based on the detection limits for each algorithm was specified. Algorithms vary in the way they calculate a detection limit, but the existence of such a threshold for the generation of an alarm is a common feature among all detection methods.

**Figure 1:**
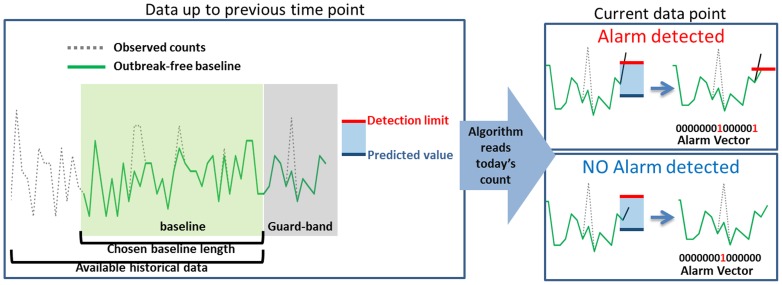
Schematic representation of the on-line process of detection of outbreak signals and correction of the observed counts series in case of alarm, in order to continually store an outbreak-free baseline.

Each algorithm was trained using *outbreak-free baseline* data constructed using data from 2008 and 2009. During prospective monitoring, on-line automated filtering was implemented by specifying that, in case of alarm, the detection limit value (minimum value that would trigger an alarm) should be stored as part of the *outbreak-free baseline*, rather than the observed value for that time point. This process is outlined schematically in Figure 1.

This process was implemented individually – in parallel – for each of the three algorithms. Real data available for the years 2009 and 2010 were used to assess, upon evaluation of plotted results, whether the correction method was successful in filtering out random peaks and potential outbreak signals. Plots were also used for qualitative evaluation of the ability of each algorithm to remain sensitive to outbreak signals after the first days of the signal had been incorporated into the baseline. Quantitative evaluation was then performed using simulated data. Using the *BLV series*, 100 years of baseline activity were simulated as described above. Two-hundred flat outbreak signals of two weeks duration and magnitude equal to three times the baseline data were injected in pairs, one outbreak pair per year. Each pair was composed of two outbreaks separated from each other by only 10 days. The percentage of outbreak signal days detected (sensitivity per day) was compared for outbreak signals which were from the first or the second in a pair.

### Combining algorithms: Scoring system

During the process of evaluating the detection limits which would provide the best balance between sensitivity and specificity of detection, for each algorithm, it was observed that no single detection limit would provide this optimum balance for all 10 syndromic series evaluated [13]. It became evident that the system should be able to operate under multiple detection limits. This was explored by maintaining several detection limits for each algorithm in all syndromic series, and using these to generate an overall score representing the “severity” of any alarm.

For each algorithm, five detection limits were implemented: the detection limit that should result in the preferred balance between sensitivity and specificity for most of the ten syndromic series evaluated, as noted above for each algorithm; and two additional detection limits above and below this initial value. The lower detection limits are more sensitive, and the higher limits more specific. The lowest detection limit for each algorithm was determined as one which would result in specificity equal to 97% in at least 6 of the 10 syndromic series evaluated [13]. The five detection limits for each algorithm are listed in Table 1.

**Table 1:** Detection limits for each of the three algorithms implemented and corresponding alarm scores.

Alarm score	EWMA*	Shewhart*	HW**
Score = 1	1.50	1.75	95.5%
Score = 2	1.75	2.00	96.5%
Score = 3	2.00	2.25	97.5%
Score = 4	2.25	2.50	98.5%
Score = 5	2.50	2.75	99.5%

= Exponentially Weighted Moving Averages control chart; Shewhart = Shewhart control chart; HW = Holt-Winters exponential smoothing. EWMA

*standard deviation.

**confidence interval.

Each algorithm evaluates the current count for the syndromic series being monitored using all five detection limits, and a *detection score* is generated corresponding to how many of these thresholds the current value has exceeded, that is, a *detection score* with a value between 0 and 5.

Combining the results from the different algorithms in this context became straightforward, as the detection scores for each algorithm could simply be added, to generate a *final alarm score* with a value between 0 and 15. Customization of detection for individual syndromic series was implemented by allowing the analyst to set an overall *reporting threshold* independently for each syndrome. That is, the analyst can manipulate the minimum *final alarm score* which will cause the system to report an alarm, by syndrome. This threshold can be changed at any time in order to increase sensitivity (using a lower threshold) or specificity (by setting a higher threshold).

Data from 2010 and 2011 were used to test the scoring system, in order to visualize the alarms generated by the system against real data streams. Then, using simulated data, system sensitivity and specificity were estimated. The scoring system was applied to over 100 simulated outbreaks of each shape, magnitude and duration, in order to calculate the sensitivity of the system using different *reporting thresholds* (1 to 15). *Sensitivity of outbreak detection* was calculated as the percentage of outbreaks detected from all outbreaks injected in the data. An outbreak was considered to have been detected when at least one outbreak day generated an alarm. *Sensitivity per day* was also calculated as the percentage of days that generated an alarm from all outbreak signal days.

The percentage of days with *false alarms* was calculated after applying the same thresholds to 35 years of simulated data which had not been injected with outbreaks. The modeled variability in syndromic counts according to month and day of the week, and the stochastic elements added by sampling values from a Poisson distribution, were assumed to mimic the natural variability in real data that would tend to generate false alarms, based on results from extensive retrospective analysis of these data streams [12].

### System reports

Once a set *reporting threshold* is reached for a given syndromic series, an alarm is generated, that is, a report is triggered. Syndromic surveillance development based on this data source has been an initiative of the data provider (the AHL) and the Ontario Ministry of Agriculture, Food and Rural Affairs (OMAFRA), responsible for the programs of animal disease surveillance in the province. A designated pathologist from AHL and a designated epidemiologist from OMAFRA are the end users of the system developed, and are referred as the “analysts”. These analysts will be responsible for receiving system outputs, interpreting them, and if necessary following up on alarms. The contents of the reports generated in case of an alarm were discussed with analysts, and the final format adopted is presented in the results. These reports were generated as PDF files, which were then automatically emailed to analysts in case of alarms. Analysts also receive reports for every syndromic series in a regular weekly email.

All methods were implemented using modules from the R environment (http://www.r-project.org/) [17], and the codes are available from the first author upon contact.

## Results

### Automated filtering potential outbreak signals

**Figure 2:**
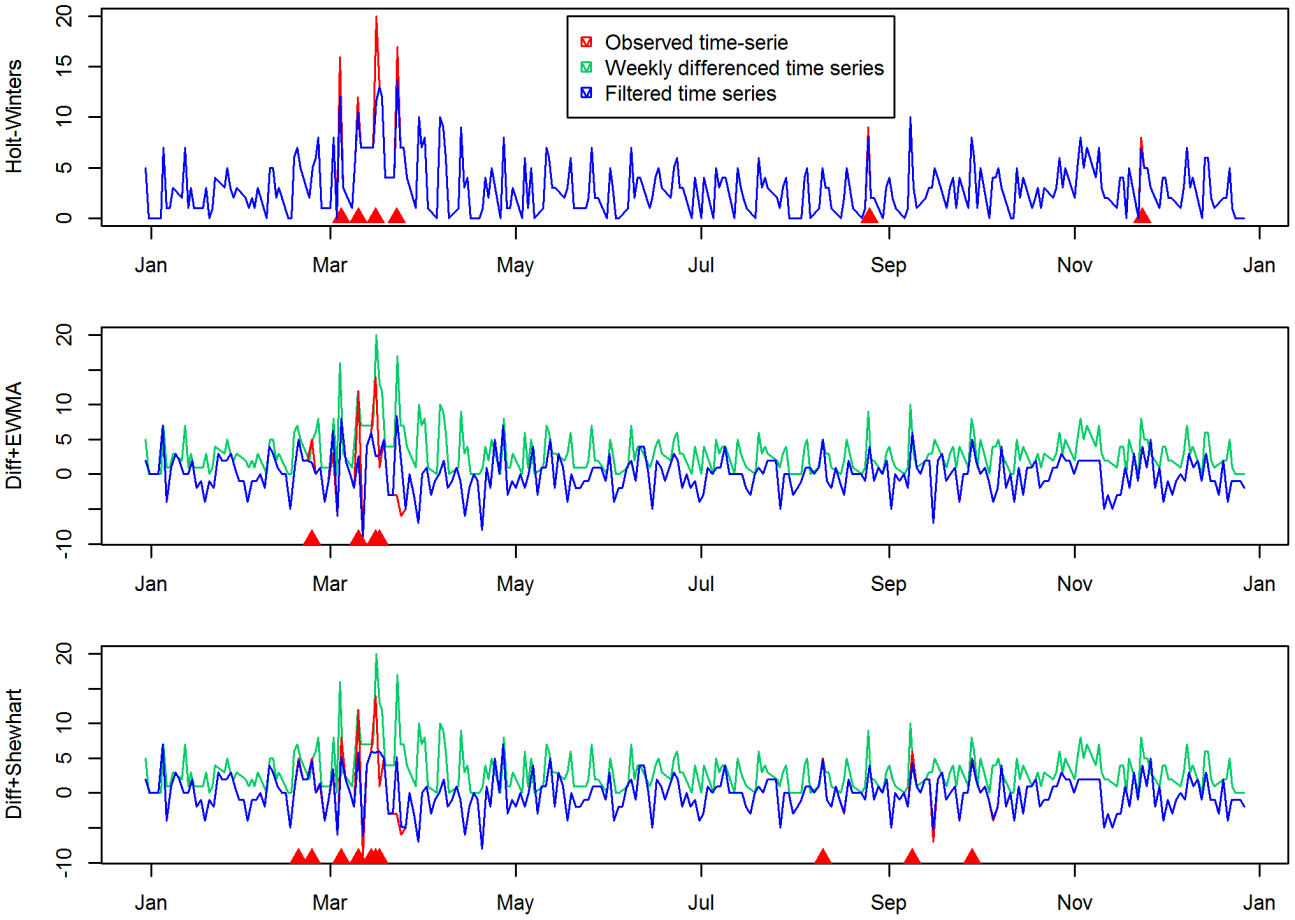
Automated correction in the BLV series for 2010 in order to remove possible outbreak-signals and excessive noise. Before attempting to use control charts the data have been pre-processed to remove temporal effects using weekly differencing. The original data are represented in green lines. The series subjected to monitoring are shown in red, superimposed on by blue lines showing the corrected series. Alarms are shown as red triangles along the bottom of each graph.

The results of filtering aimed at preserving the outbreak-free baseline are shown in Figure 2 for the *BLV series* in 2010. For the control charts the series subjected to monitoring is composed of the residuals of weekly differencing (applied to remove temporal effects), rather than the observed time series, which are shown in green. The results indicated that automated filtering using the detection algorithm was effective. Besides a visual comparison between the original and the corrected time series, this conclusion is based on the fact that all algorithms were able to flag outbreak signals in multiple days, even past the guard-band period (10 days). This implies that the outbreak signals observed were not incorporated to the baseline, and that the algorithms remained sensitive to consecutive outbreak signal days.

This qualitative analysis was followed by quantitative comparison of the detection performance in outbreaks signals injected in pairs. When the automated filtering was not implemented, the reduction in sensitivity per day during the second outbreak signal was 24.3% for the Shewhart control chart, 14.9% for the EWMA control chart, and 8.8% for the Holt-Winters exponential smoothing. When filtering was implemented, these differences were reduced to 12.6%, 6.2% and 3% respectively. In addition to the smaller reduction in sensitivity, the HW correction approach presents two qualitative advantages over the other two methods. First, the results are simpler to interpret. Since the time series is not altered by differencing, analysts can readily compare the filtered series with the original observed counts, in order to qualitatively assess the performance of the detection algorithm in filtering out random spikes and potential outbreak signals. Second, because this algorithm can deal with the temporal effects present in the data, its detection limits for each time point reproduce these effects.

The implementation of a combined scoring system allowed the three detection algorithms to be implemented in parallel. However, for their results to be combined sensibly it was considered essential that the algorithms were operating under the same conditions, that is, that they were using the same baseline. If automated filtering was implemented in parallel using all three algorithms, as time passed, and most especially in case of repeated outbreak signals, each of them would effectively utilise different baselines. Based on the conclusions presented above, HW exponential smoothing was selected as the sole method to preserve the outbreak-free baseline by automated filtering of the data streams.

### Scoring system

#### Detection using real data: qualitative analysis

**Figure 3:**
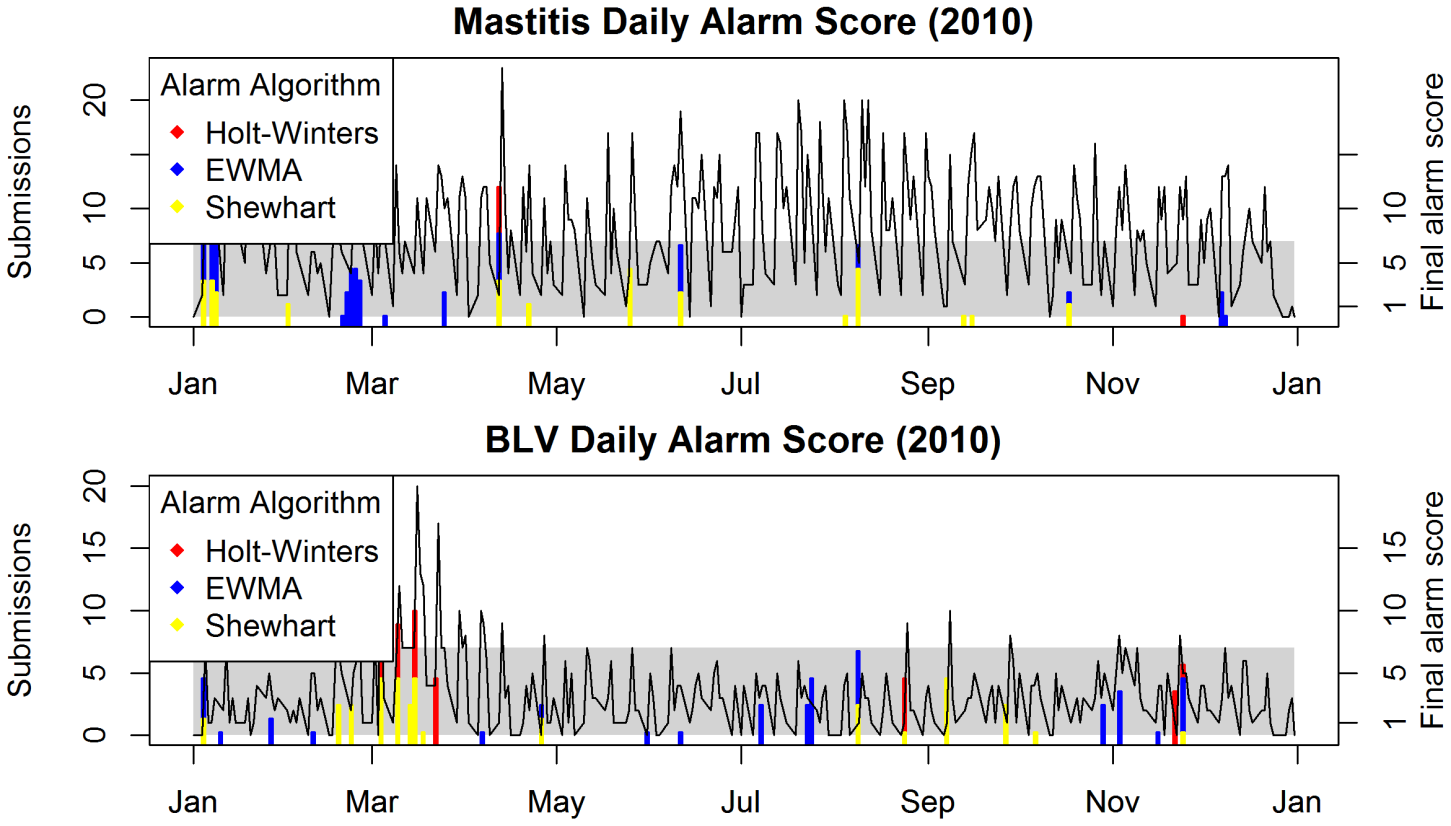
Outbreak-signal detection using three algorithms (Shewhart control charts, EWMA control charts and Holt-Winters exponential smoothing) combined using the scoring system and applied to real data. The top panel plots the Mastitis series for the year 2010. Detection scores for each algorithm are shown as vertical bars, stacked to give a final alarm score which scale is shown in the secondary axis. The gray rectangle is used to mark the limit in the secondary axis which corresponds to the reporting threshold – here 7. The bottom panel shows a similar graph for the BLV series.

Figure 3 shows the results of applying the scoring system to 2010 data, for the *mastitis* and *BLV* syndrome series. In this figure a reporting threshold of 7 for both syndromes was used to illustrate the method. That is, the analyst will only receive a report when the vertical bars representing the summed detection scores for all algorithms (*final alarm score*) *is equal or greater than* 7 (and therefore is higher than the grey shaded area, which limit was set to 6.5). This would have happened only once, in April, for the *mastitis series*, and on three occasions, likely related to the same ongoing process in March, for the *BLV series*. Visual evaluation of detection performance is difficult due to the day-of-week effects in the data, which can be misleading when judging the presence of unexpected peaks in counts. True quantitative analyses are reported below.

Application of the scoring system to real data was an important step in evaluating how the system might add value to the analysis performed. The analyst can, by looking at the graphs illustrated in Figure 3, compare the final alarm score to the information regarding the behaviour of the data. The analyst will also know which algorithms were responsible for the alarm signal, and the individual scores generated. For the *BLV series*, for instance, small absolute signals between July and September indicate that some days with seemingly normal activity resulted in the generation of detection signals by the EWMA algorithm; likely indicating that these observations were somewhat unusual for that day-of-week. For the *mastitis series* a generally higher concentration of test requests can be observed between July and September. However, the detection scores indicate that these were not highly unexpected, with only a few, low level, detection signals being generated by the control-chart algorithms. The HW algorithm, which can account for temporal effects more explicitly, did not generate any detection signals during this period, leading to the hypothesis that the generally increased numbers may be a temporal effect, such as a seasonal trend. It is also evident that lowering the *reporting threshold* of the *mastitis series* to 6, for instance, would have generated a much larger number of alarms in the 2010 syndrome data stream. These would arguably be false alarms, as no outbreak has been documented in the province in that year. Eight other syndromes monitored as part of this research were evaluated (graphs not shown), and three of these required adjustment of their combined *reporting threshold* to a value of 9 or 10 in order to prevent excessive numbers of false alarms.

#### Detection using simulated data: quantitative analysis

**Figure 4:**
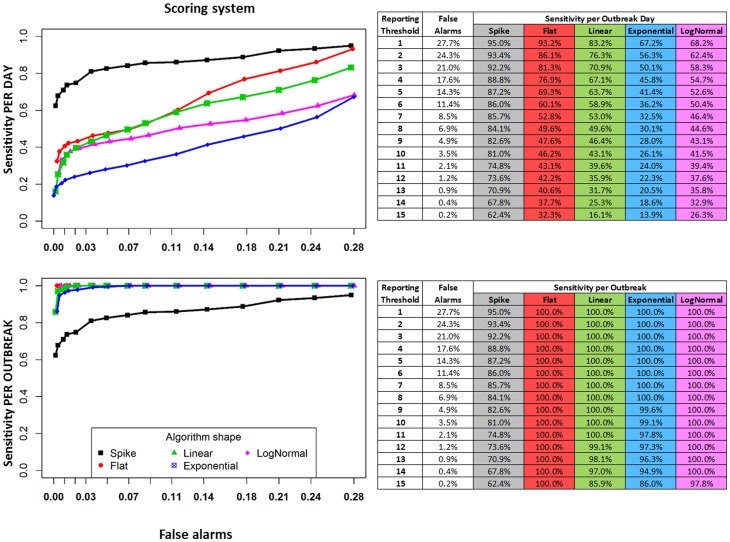
Sensitivity of detection and false alarm rates when the combined algorithms are applied to the simulated mastitis series, with five different shapes of simulated outbreak signals. The table rows and graph nodes show different final alarm scores used as the reporting threshold. Values in the table correspond to the median among 3 different outbreak magnitudes (1 to 4 times the background activity of the series) and 3 different outbreak lengths (1, 2 and 3 weeks; except for the spike, which is always one single day).

Figure 4 shows the results of applying outbreak signal detection using the scoring system against the simulated *mastitis series*. As many scenarios were evaluated, only the median performances are shown; the graph's purpose is to highlight the comparative performance of system settings across a range of outbreak shapes. The results indicate that decreasing the reporting threshold of the scoring system can result in great sensitivity, but at the cost of higher levels of false detection.

**Figure 5:**
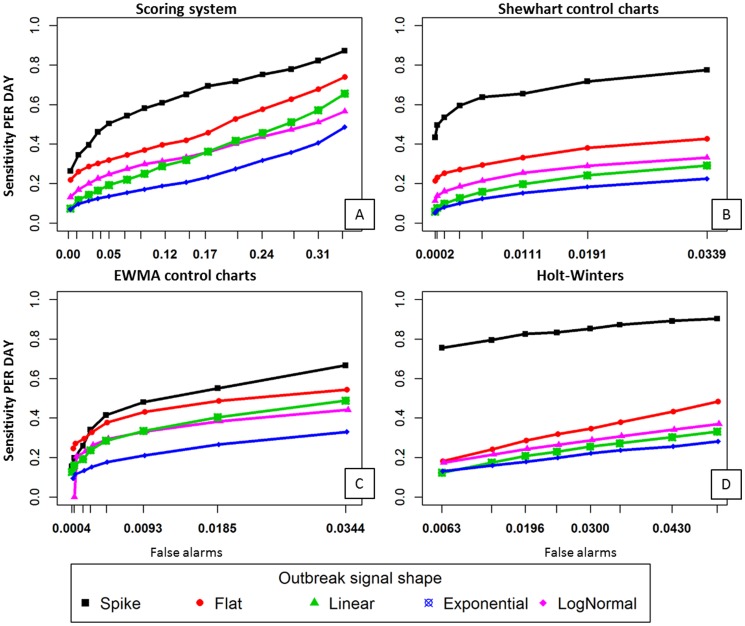
Sensitivity of detection and false alarms rate in the BLV series. Panel A shows the sensitivity of detection compared to false alarms rate when all three algorithms combined using the scoring system are applied to the simulated BLV series, with five different shapes of simulated outbreaks injected. The graph nodes show different final alarm scores used as the reporting threshold. Points represent the median among 3 different outbreak magnitudes (1 to 4 times the background activity of the series) and 3 different outbreak lengths (1, 2 and 3 weeks; except for the spike, which is always one single day). The remaining panels show sensitivity and false alarm when each detection algorithm is applied individually, as previously documented in [13].

Besides high sensitivity, an advantage of using multiple algorithms is shown when the detection is compared to individual algorithms for each shape of outbreak signal. Figure 5 compares the results associated with sensitivity and false alarms in the *BLV series* by contrasting the performance of the combined approach with the individual algorithms as documented in previous work [13]. The HW algorithm, for instance, showed higher sensitivity than the scoring system for the detection of spike signals (black lines, sensitivity plotted in the y-axis), but the sensitivity achieved by this algorithms is lower than the sensitivity of the scoring system, in the ranges of detection limits investigated, for all other outbreak signal shapes. A similar result can be observed for the Shewhart control chart, with the detection of spikes showing high sensitivity with the algorithm, but the sensitivity of detection of other shapes being higher when the scoring system is applied. The comparisons must be made with caution, taking into account the differences in the range of false alarms observed with the different approaches. The scoring system is capable of reaching sensitivity up to two times higher than the EWMA algorithm for all algorithm shapes, in the range of detection limits evaluated, but the number of false alarms is also higher than for that algorithm.

Overall, Figure 5 illustrates that the use of multiple algorithms in combination allows the system to achieve higher sensitivity for a range of outbreak types, as outbreak shapes not detected by one algorithm will be detected by another. Once again as the main purpose of these graphs is to provide the analyst with a tool to compare across algorithmic approaches and outbreak shapes only median values are shown in Figure 5.

### System reports

**Figure 6:**
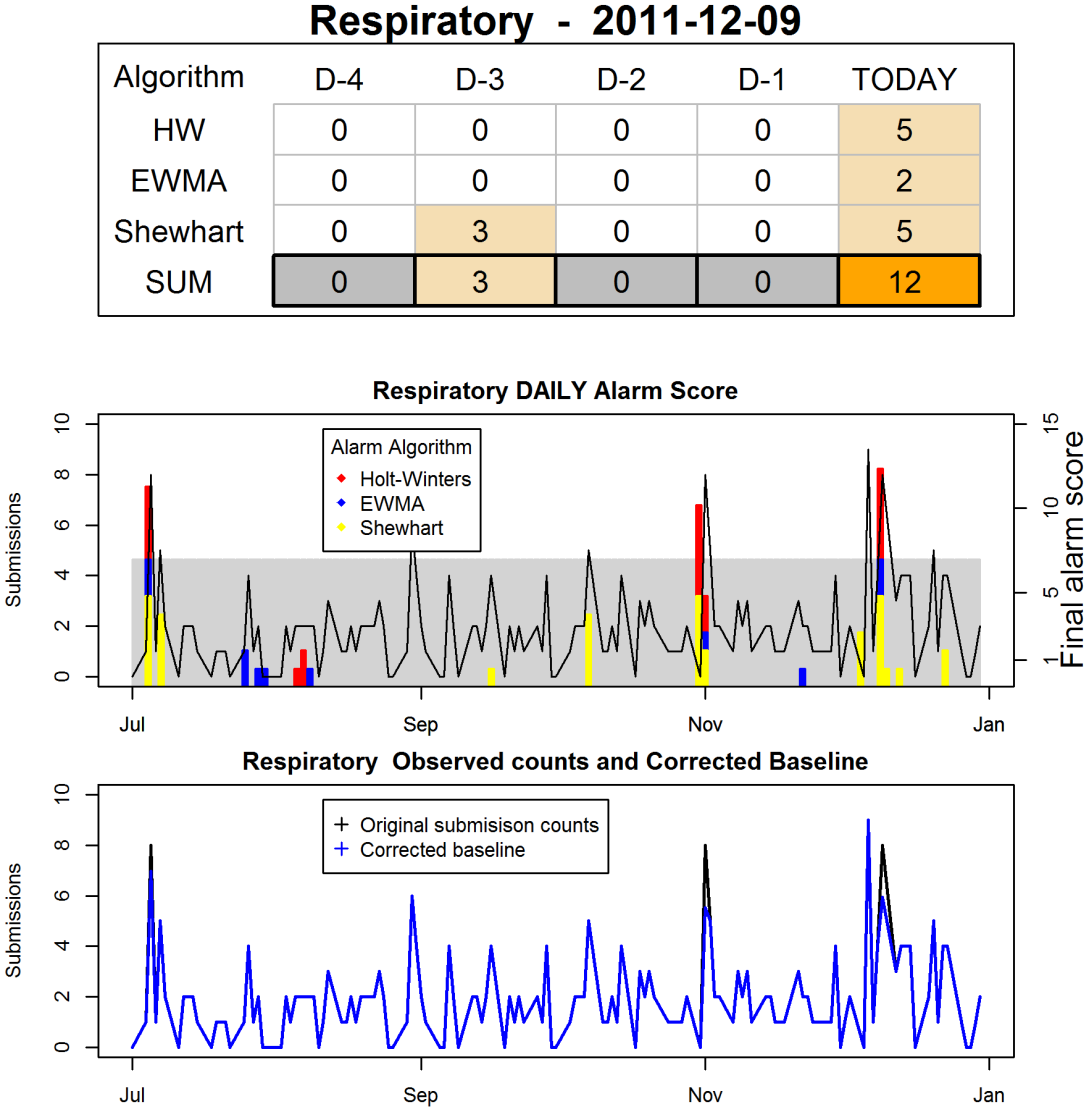
Example page of a daily report sent to analysts in case of alarm. The top table shows the detection score for the three algorithms used, in the last 5 days. Next the data of the last 26 weeks are plotted against the detection score for all three detection algorithms used, stacked to give a final alarm score. The main y-axis is the scale for the data, and the secondary y-axis gives the scale for the detection scores. The gray rectangle shows the range of final alarm score which will not generate an alarm. The bottom panel shows the observed data, superimposed by the data after outbreak-signal removal by the detection algorithm.

In the event of an alarm, the analyst receives an e-mail with an attached PDF file. The first page of the file contains the list of all the syndromes being monitored, with all those for which an alarm has been generated on the given day highlighted in red. Individual reports for the syndrome(s) which generated alarm(s) follow on individual pages. An example report page is shown in Figure 6. This report was generated because the *final alarm score* for the Respiratory series was 12, against a defined *reporting threshold* of 7. In the top panel, the analyst can see the *final alarm score* for the current day, which shows why the report was generated. The analyst can also quickly glance at the previous 4 days (one full week, since 5-day weeks are used in the system). From the lower panels the analyst gains a broader view of the data behaviour, as well as detection algorithm performance, over the last 6 months. In the middle panel *detection scores* are plotted as a secondary axis, and the *reporting threshold* is shown as a gray box in the background. The bottom panel allows the analyst to assess visually the performance of the automated filtering to preserve an outbreak-free baseline.

## Discussion

The role of laboratory data in the rapid detection of outbreaks has been recognized in public health, partly due to the extensive area coverage provided by these data in comparison to clinical data coming from individual practitioners or hospitals [18]. In a series of steps we have developed methods and a system to implement syndromic surveillance in animal health based on veterinary laboratory data. Having concluded that laboratory test requests represented an opportunistic data source with great potential for syndromic surveillance systems in livestock medicine [19], we explored diagnostic submissions for cattle made to the Animal Health Laboratory in the province of Ontario, Canada, in order to construct a monitoring and early disease warning system for that province.

Steps to classify data into syndromes [11], and to evaluate these data retrospectively so they could be prepared for monitoring [12], were documented. Using the data available together with simulated data, the performance of different detection algorithms was evaluated [13]. This indicated that algorithm performance depended on the shape of the outbreak signals encountered, as well as the baseline characteristics of each individual syndromic series being monitored.

In the current paper, the implementation of multiple algorithms in parallel has been explored, together with the challenge of preventing outbreak signals from contaminating the training data set. The latter goal was addressed first. The correction of baseline series in case of alarms has mainly been discussed for regression methods [9], [10], and it is generally based on preventing model parameters from updating in cases where an alarm has been generated. The use of a guard-band between the baseline data and the time point being evaluated [4], [3], [8] can avoid incorporation to the baseline of an outbreak signal before its first detection, but it does not provide a method to maintain an outbreak-free baseline. In the present work the detection limit of each algorithm is used to correct the observed data, continuously storing an *outbreak-free baseline* which is used by the algorithms as training data. The method proved effective for all three algorithms explored, however, Holt-Winters exponential smoothing was chosen due to its advantages in terms of interpretability and explicit modeling of temporal effects in the data.

A detection system should be able to detect a variety of outbreaks with different signatures [20], [21]. This is especially important when the outbreak signature is not known. However, different detection algorithms typically demonstrate optimal performance for outbreaks with a specific temporal progression pattern; a challenge if one specific algorithm has to be selected [6]. The use of multiple algorithms in parallel has been explored through the use of decision rules which pool the binomial results from different algorithms [6], or by using goodness-of-fit tests to decide when to switch between algorithms [21].

This work combined three algorithms that had demonstrated solid performance in detecting outbreaks signals of varying shapes across a range of syndromes which had been subjected to monitoring [13]. These algorithms were: Shewhart control charts designed to detect sudden spikes; EWMA control charts developed to detect slow increases in counts; and Holt-Winters exponential smoothing which can explicitly account for temporal effects. For each algorithm, multiple detection limits were used, in order to transform the outcome of each method into a magnitude score, rather than a binomial signal indicating whether a potential outbreak signal was present or not. These detection scores were then combined to produce a final alarm score. All algorithms contribute to the measure of alarm magnitude, and this combined magnitude is used to decide whether analysts should receive an alarm report or not. In case of any alarm analysts can review the output of all three detection algorithms across the range of monitored syndromes.

The use of magnitude scores, rather than a binary alarm decision, results in the analyst being responsible for the definition as to when an alarm will be triggered. This is seen as a positive feature. Considering the number of external factors that can influence fluctuations in the data being entered into any syndromic surveillance system it is expected that, once an alarm has been raised, a human analyst will review the output in the light of relevant factors and decide whether a true problem exists [22]. This is even more critical in animal health data than in the human case, since laboratory submission is not just a function of disease but also of animal value [23], and several economic factors have been associated with the rate of diagnostic submission to laboratories [24].

It becomes critical, therefore, to develop system outputs that provide as much information as possible, according to the capabilities of the data at hand and the system. This was addressed by developing output charts that combine observed data with the detection scores for all three algorithms, plotted over time, and provided frequently to the analyst. Although the charts combine a lot of information, the consistency of the presentation results in rapid familiarization. Once the analyst becomes familiar with their interpretation, the frequent inspection of reports should inform the analyst in the behaviour of the data and algorithms. For this reason the monitoring results for all syndromes are emailed to analysts weekly, regardless of the detection of any signal. Should an alarm be detected, the analyst will be able to judge, based on the past behaviour of the data, whether to challenge that alarm.

The use of algorithms that can detect various outbreak signal shapes, coupled with a user interface that allows flexible customization of alarms, resulted in a robust system. Multiple syndromic series are analysed within the same process, even if the expected patterns of disease spread would be different in cases of outbreaks for each of those syndromes. This means that in the future more syndromes can be incorporated into the system without the need to implement additional detection algorithms or to make significant changes to the statistical analyses underlying the system. The statistics which comprise the core of the system need not be changed once the system is in place. The end user (the analysts) can fine tune to system to increase performance individually for each syndrome. All the work described here for fine tuning (for instance increasing the reporting threshold of some syndromes in order to avoid false alarms) has been designed to mimic the natural manner in which the system would adjust to analysts' needs after one year of use.

While analysts will review the system outputs, taking information regarding the detection scores of each algorithm into consideration, and make the final decision regarding any alarm, it is not expected that all system users will have sufficient background in quantitative methods to decide which algorithms are best for each syndrome. Their decisions regarding changes in the reporting thresholds that generate alarms will be mainly operational, that is, “is the current number of alarms generated manageable”. This is the reason why a combined score, rather than a choice of different algorithms for individual syndromes, was considered more robust and easier to interpret and manipulate by the end users. User tweaking, however, cannot actually degrade system performance. It should be remembered that while analysts can change the reporting thresholds, they do not adjust core parameters associated with any of the algorithms – e.g., the detection limits applied to the data streams or the thresholds which trigger data correction to filter out outbreak signals and excessive noise. Thus the performance of the algorithms is expected to remain unchanged regardless of the choices made by the analysts concerning reporting. In addition the default weekly generated reports ensure that raw rates of syndrome observation remain transparent. Thus if an analyst were to drastically reduce the sensitivity by setting excessively high reporting thresholds it is likely that the problems associated with this choice would be obvious to those using the system. In addition, because automated filtering will have continued as the system runs, the outbreak-free baseline will have incorporated outbreak signals, which are filtered out even if a reporting threshold was not reached.

In evaluating the performance of a syndromic surveillance system when applied to historical data, it is generally difficult to estimate its performance due to lack of documentation as to the causes of the extraneous signals registered in the data [18], such as those seen in the *BLV series* in Figure 3. However, the continuous inspection of system outputs should enable analysts to progressively tailor the system for optimum performance. The system described here allows for a high degree of customization by the analyst, who can change the reporting threshold that triggers an alarm individually for each series, according to the observed behaviour of the algorithms, or to comply with institutional objectives. For instance, if too many false alarms are being observed for a specific syndrome, the reporting threshold for that individual syndrome can be raised, in order to increase specificity. Reporting thresholds can be set high in order to generate fewer reports; perhaps limiting analysis to the inspection of the regular weekly reports. If a specific syndrome required more intensive monitoring, the threshold could be lowered to increase sensitivity.

The choice to combine all three algorithms, however, came at the cost of a decreased specificity for the system as a whole (slightly higher rates of false alarms), which is expected behaviour when multiple diagnostic tests are applied in parallel [25]. Surveillance systems based on laboratory data in general should prioritize sensitivity and timeliness over specificity, since the coverage of laboratory data is small (that is, “small increases in laboratory data often indicate larger communitywide out-breaks” [18]).However, it has also been highlighted that this increase in sensitivity should not result in an unmanageable number of signals [18]. Despite the higher percentage of false alarms identified when using simulated data, the results of applying detection based on the scoring system showed that the number of detected outbreaks was never greater than 4 per year for any of the 10 evaluated syndrome series providing that the individual reporting thresholds were optimized for each syndrome. With continuous system optimization, the number of false alarms is expected to decrease [18], without any significant loss in system sensitivity.

The potentially high number of false alarms during the initial phases of system implementation, as well as the need for continuous inspection of system reports and parameters optimization, have been key points of contention in an ongoing debate regarding the value of syndromic surveillance [26]. After comparing syndromic surveillance results to outbreaks detected locally by traditional surveillance van den Wijngaard et al. recommended “the use of syndromic surveillance to reveal blind spots of traditional surveillance”, as well as for “monitoring disease burden and virulence shifts of common pathogens” [26]. The system discussed here, developed using laboratory submission data to the AHL, will serve as a backup to traditional animal health surveillance in the province of Ontario, detecting outbreaks that are widespread across the province or which are evolving too slowly to be noticed by clinicians or pathologists. Moreover, the second recommendation made by van den Wijngaard et al.[26] is a key feature of this system, with regular compilations of observed data being delivered to analysts, which will contribute to situational awareness in animal health surveillance.
